# Integrating Worker and Food Safety in Poultry Processing Through Human-Robot Collaboration: A Comprehensive Review

**DOI:** 10.3390/foods15020294

**Published:** 2026-01-14

**Authors:** Corliss A. O’Bryan, Kawsheha Muraleetharan, Navam S. Hettiarachchy, Philip G. Crandall

**Affiliations:** Food Science Department, University of Arkansas, 2650 Young Ave, Fayetteville, AR 72701, USA; cobryan@uark.edu (C.A.O.); nhettiar@uark.edu (N.S.H.)

**Keywords:** human-robot collaboration, poultry processing, worker safety, food safety

## Abstract

This comprehensive review synthesizes current advances and persistent challenges in integrating worker safety and food safety through human-robot collaboration (HRC) in poultry processing. Rapid industry expansion and rising consumer demand for ready-to-eat poultry products have heightened occupational risks and foodborne contamination concerns, necessitating holistic safety strategies. The review examines ergonomic, microbiological, and regulatory risks specific to poultry lines, and maps how state-of-the-art collaborative robots (“cobots”)—including power and force-limiting arms, adaptive soft grippers, machine vision, and biosensor integration—can support safer, more hygienic, and more productive operations. The authors analyze technical scientific literature (2018–2025) and real-world case studies, highlighting how automation (e.g., vision-guided deboning and intelligent sanitation) can reduce repetitive strain injuries, lower contamination rates, and improve production consistency. The review also addresses the psychological and sociocultural dimensions that affect workforce acceptance, as well as economic and regulatory barriers to adoption, particularly in small- and mid-sized plants. Key research gaps include gripper adaptability, validation of food safety outcomes in mixed human-cobot workflows, and the need for deeper workforce retraining and feedback mechanisms. The authors propose a multidisciplinary roadmap: harmonizing ergonomic, safety, and hygiene standards; developing adaptive food-grade robotic end-effectors; fostering explainable AI for process transparency; and advancing workforce education programs. Ultimately, successful HRC deployment in poultry processing will depend on continuous collaboration among industry, researchers, and regulatory authorities to ensure both safety and competitiveness in a rapidly evolving global food system.

## 1. Introduction

The global food processing industry plays a vital role in transforming raw agricultural products into safe, convenient, and shelf-stable foods. With continued population expansion and rising consumer demands for diverse, ready-to-eat options, food processing has become an increasingly indispensable sector worldwide. However, this growth also brings significant challenges, specifically regarding the safety of both workers and end consumers [1].

Poultry processing in the broader food industry stands out for its high production volumes, fast-paced workflows, and susceptibility to microbial risks, particularly *Salmonella* and *Campylobacter* [2]. According to projections by the Organization for Economic Co-operation and Development (OECD) and the Food and Agriculture Organization (FAO), global poultry production is expected to rise by 16% by 2033 [3], further amplifying these risks. Because hazards are simultaneous (ergonomics, machine guarding, chemicals, line speed, human factors), safety cannot be managed with single fixes in isolation. Instead, an integrated safety program treats the plant as an interacting system; physical layout, equipment design, work organization (including line speeds and breaks), training and language access, maintenance practices, data/metrics, and regulatory compliance are all linked [4]. Recent research demonstrates that environmental education interventions, even over short time frames, can create lasting improvements in safety-related consumption habits, particularly in vulnerable communities. In the context of poultry processing, cultivating a ‘consumption of safety’ through targeted educational outreach, addressing everything from hand hygiene and equipment cleaning to safe storage and cooking, has the potential to reduce contamination risks and foodborne illnesses. Establishing a robust safety culture, with buy-in from workers, managers, and consumers alike, transforms safety from an imposed rule set into a shared value and everyday practice [5].

The search for solutions to enhance safety has driven the increasing adoption of automation in food processing. Collaborative robots (cobots) have attracted attention for their flexible, human-centric design, which enables them to work side by side with human operators, sharing a common goal [6]. Potential benefits of cobots include removing workers from the most dangerous, repetitive, or precision tasks (e.g., deboning, repetitive sorting, heavy lifting), and reducing cuts, amputations, and MSD risk. They may also improve consistency and product traceability (software + sensors), reducing unsafe human improvisation and speed-related pressure. Automation vendors already offer robotic deboning/packing systems for poultry lines [7].

Studies and reviews show automation has mixed outcomes: while robots can reduce some hazards, they may shift risks (e.g., new maintenance hazards, complex human–robot interactions, increased line speeds that re-expose workers to faster cycles). Evidence from recent reviews and industry reports warns about trade-offs and the need for careful evaluation [8]. The social context matters because workers in poultry plants are often immigrants, low-wage, and may fear losing jobs. Poor management changes can reduce reporting and mask safety problems even after new technology is installed. Recent reporting and USDA work note persistent high MSD risk despite automation [9].

This human-robot collaboration (HRC) is a growing area of focus in the food sector. Researchers are now exploring ways to integrate human skills, such as decision-making and critical thinking, with robots’ strengths (repeatability and accuracy) to perform complex tasks [10]. The poultry processing industry has not yet adopted robotics at the product level, relying heavily on human workers to debone chickens to achieve the maximum meat yield [11].

## 2. Methodology

The databases Web of Science, PubMed, ScienceDirect, SpringerLink, and Scopus were searched for literature on collaborative robots using the following keywords: human–robot interaction (HRI), cobots, AI in robots, collaborative learning, and HRC, with poultry processing as a subheading. Peer-reviewed academic journals, conference papers, and reviews published since 2018 and written in English, and those that contain qualitative or quantitative information or both, were included in this systematic review. We also searched gray literature using these same terms. We initially identified 156 articles on collaborative robots and their applications through database searches. Out of 156 articles, 30 duplicate papers were removed. By screening the remaining 126 papers, 40 records were excluded based on title and abstract. A total of 86 full-text articles were considered for eligibility, of which 43 were excluded for various reasons: publications before 2018, not relevant to cobots, AI techniques not addressed in the study, and inconsistent results (the results were not the same throughout the manuscript). Finally, we included 43 full-text articles published between 2018 and 2025 for review.

## 3. Current Challenges in Poultry Processing

Poultry processing plants feature high-speed production lines, cold, wet environments, and repetitive tasks, which pose a risk of various occupational hazards [12]. Employees frequently perform repetitive motions, such as trimming, packing, or operating machines, which can lead to chronic musculoskeletal disorders or acute injuries [13]. Moreover, extended shifts and physically demanding tasks contribute to fatigue, leading to higher incident and injury rates [12].

### 3.1. Worker Safety Concerns

Common injuries include repetitive strain injuries (RSIs), cuts, lacerations, and slips or falls, underscoring the inherently risky nature of many food processing jobs [14]. Regulatory bodies, both internationally and in the United States, have established guidelines to minimize these risks through safe equipment operation, ergonomic assessments, and stringent training requirements. FSIS has studied the effects of line speed on work-related musculoskeletal disorders. However, line speeds were not identified as the primary factor contributing to the risk of musculoskeletal disorders among workers at these plants. However, insights were presented into how line speeds and other factors may contribute to employees’ overall risk [15]. Among the recommended steps was increasing staffing, thereby lowering the per-worker piece rate. Other suggested strategies include effective ergonomics plans, knife-sharpening programs, and medical management. The reports indicate that meat and poultry companies have developed an industry-wide set of best practices, reflecting input from both workers and companies.

### 3.2. Food Safety Risks

The frequency of *Salmonella* and *Campylobacter* in raw poultry increases food safety risks [2]. Any lapses in sanitation, equipment cleaning, or employee hygiene can lead to cross-contamination and potential outbreaks. High line speeds and manual handling further compound these risks, as rapid workflows increase the probability of mistakes, missed cleaning schedules, or improper handling procedures. Consequently, poultry recalls and contamination incidents, although not unique to this sector, often garner significant public attention due to poultry’s widespread consumer base [16].

The line between worker safety and food safety in poultry processing can be surprisingly thin. Manual production in the food industry inherently poses risks to worker health, including repetitive strain injuries and exposure to less-than-ideal working conditions. These issues frequently intersect with food safety concerns, such as poor hygiene and cross-contamination. For example, manual handling increases the likelihood of cross-contamination from surfaces exposed to pathogens, while poor ergonomics or fatigue could lead to oversights in cleaning and sanitizing. This intersection highlights why a holistic view of safety, one that addresses both human health and product hygiene, is essential for long-term sustainability and consumer trust.

### 3.3. Limitations of Traditional Automation

Specialized machines already exist that automate specific parts of the meat processing workflow, such as forming machines that shape 200,000 nuggets per hour [17]. However, these technologies are not adaptable or collaborative. For example, Liu et al. [18] and Xie et al. [19] used 2D cameras for image segmentation and planning cuts on sheep and pork carcasses. Wang and Cai [20] used contact-force feedback to adjust the cutting speed and cutting tool angle during cutting pork carcasses. Specific parts of the process are automated, and the robot operates in an isolated space from the workers. Many current technologies are designed with a single, specialized function in mind [21] and have historically been implemented in ways that substitute for human labor rather than enabling humans and machines to work together [22].

To drive widespread adoption of poultry automation in small and mid-sized processing plants, adaptive technologies must prioritize two critical challenges:

1. Carcass Variability: Systems must intelligently adjust to bird size, shape, and quality differences without sacrificing efficiency.
2. Dynamic Workflows: Automation should integrate real-time data to respond to shifting line speeds, equipment hiccups, or changing production demands.

By embedding flexibility into robotic systems and AI-driven tools, smaller facilities can achieve the precision of large-scale operations while maintaining cost-effectiveness. Solutions like modular robotics and vision-based sorting algorithms already show promise; scaling these innovations will democratize automation across the industry [23].

## 4. Technological Innovations in Human-Robot Collaboration

### 4.1. Robotic Systems for Poultry Processing

The successful integration of collaborative robots (cobots) into food processing, particularly in poultry processing, depends heavily on establishing worker and food safety standards. While automation with collaborative robots promises a safer, more efficient workflow, any deviation from these regulations can undermine worker well-being and product quality.

Industrial robotic arms are widely used in the food industry as manipulators for pick-and-place operations, offering high positional accuracy and rapid motion. These systems can increase efficiency, precision, and consistency in handling repetitive tasks that traditionally relied on human labor [24,25]. However, the adoption of industrial robotic arms is often limited by significant initial investment, ongoing maintenance costs, and the need for customization, especially for small and medium-sized enterprises [26]. Robotic grippers, end effectors, cameras, and sensors that operate in the vicinity of poultry products must be designed for high-pressure water washdown and sanitation operations. These requirements impose additional cost constraints on poultry handling robots.

Among robotic end-effectors, suction cups are the standard tools used in food processing environments. They are favored for their low cost, ease of use, and effectiveness in handling items such as vegetables and eggs [25,26]. Despite these advantages, suction cups face notable limitations. They are ineffective for handling food products with moist or porous surfaces, or for irregularly shaped or fragile items, as maintaining a reliable vacuum seal becomes challenging [26]. Consequently, such tasks often rely on human workers, highlighting a significant gap in automation capabilities. Food products are generally non-rigid, often delicate, and easily damaged or marred when they come into contact with hard surfaces. Additionally, they are prone to bacterial contamination and are significantly influenced by environmental factors such as temperature, humidity, and pressure [27]. The characteristics of food products present numerous challenges when developing robotic systems to handle them.

Traditional methods of assessing food quality include Brix measurements to determine fruit sweetness, titrations to measure acidity, and manual evaluation of meat marbling. These methods are labor-intensive, slow, and destructive [28]. Optical sensors capture valuable information by analyzing the interactions of light with food. Various techniques, including reflectance, fluorescence, and Raman scattering, can be used to assess key quality indicators. Assessing meat quality is commonly done through visual inspection or sensory testing, which requires trained personnel and is both expensive and time-consuming [29]. In addition, there may be variability between the inspectors. Interpreting this data remains a challenge; however, machine learning could play a valuable role in addressing it. Researchers can extract meaningful patterns from complex datasets using AI algorithms, enabling real-time, high-precision food quality assessment [29].

### 4.2. Safety Protocols for HRC

#### 4.2.1. ISO and ANSI Standards for Robotic Systems

ISO 10218 (Parts 1 and 2) [30,31] provides comprehensive baseline requirements for industrial robot manufacturers and integrators, covering risk assessment procedures, safeguarding measures, and emergency stop capabilities (OSHA). However, the growing adoption of human-robot collaboration led to the publication of ISO/TS 15066 in 2016 [32], which explicitly addresses physical interaction between robots and humans, including maximum permissible force and pressure levels in shared workspaces. These two standards work together, where ISO 10218 focuses on general robot design and safety, initially conceived for industrial robots operating behind safety fences. ISO/TS 15066 expands on this by specifying metrics such as “maximum quasi-static force” and “maximum transient force” thresholds, which are critical for ensuring that contact incidents do not harm human operators.

In the United States, ANSI/RIA R15.06 [33] aligns closely with ISO 10218 requirements, adapting them to the American regulatory landscape (ANSI and OSHA). Also, ISO/TS 15066 has been nationally adopted as RIA R15.606 (TR 606) for Collaborative Robot Safety. Facilities adopting cobots often leverage these norms to design safety protocols, including controlled robot speeds, safeguarded zones, and advanced sensing technologies (e.g., machine vision, light curtains). For instance, a poultry deboning line utilizing cobots for repetitive cutting tasks would need to comply with force-limiting parameters to prevent the risk of laceration if a human arm were to enter the robot’s path inadvertently. This force limit is 150 N for shoulder impact, and approximately 50 N in a forearm impact. Additionally, ISO/TS 15066 restricts the linear displacement of cobot manipulators to 0.25 m/s per axis. These force and movement restrictions are generally slower than the maximum levels achieved by an experienced poultry worker. Consequently, there are concerns about the productivity levels of poultry processing lines when robotic machinery is collocated with human poultry workers. Table 1 compares these rules.

#### 4.2.2. OSHA Regulations

In parallel, the U.S. Occupational Safety and Health Administration (OSHA) offers a broad framework under 29 CFR 1910 (OSHA) that governs machinery guarding, hazard communication, ergonomics, and lockout/tagout procedures. While OSHA does not have a cobot-specific standard, it enforces overarching rules that require employers to evaluate and mitigate hazards [34]. For instance, poultry facilities deploying cobots must demonstrate that emergency stops are easily accessible, that pinch points are minimized or eliminated, and that workers are adequately trained to operate in a semi-automated environment. Failure to comply can result in citations and financial penalties, emphasizing the seriousness of worker protection in industrial settings.

### 4.3. Sanitation and Hygiene Automation

#### 4.3.1. Sanitation Standard Operating Procedures (SSOPs)

From a food safety perspective, Sanitation Standard Operating Procedures (SSOPs) outline mandatory cleaning and sanitation protocols that must be followed before, during, and after processing shifts [35]. These protocols ensure that machinery, whether fully manual or robotic, remains free from pathogenic microorganisms. In poultry processing, SSOPs often specify frequencies for hot-water rinses, alkaline foam cleaning, and disinfection cycles using chemical agents (e.g., peracetic acid or quaternary ammonium compounds). Poultry automation equipment must meet the highest international standards for protection against fluids and solid particulates entering the equipment, as well as the requirements for electrical and electronic equipment. Poultry processing equipment must meet IPC 69K requirements for resisting high-temperature fluid and particulate intrusion into electrical and electronic equipment [36]. When cobots are introduced, these procedures must be adapted to accommodate robot arm assemblies, joints, and end-effectors.

#### 4.3.2. HACCP and Regulatory Oversight (FDA, USDA)

The Hazard Analysis and Critical Control Points (HACCP) framework requires systematic identification and control of potential contamination points [37]. In poultry processing plants, critical control points often include evisceration, deboning, and packaging stations. If cobots perform any of these tasks, their design and operating parameters must comply with HACCP plans, such as maintaining a constant temperature zone or ensuring no cross-contact between raw and cooked products.

In the United States, the USDA’s Food Safety and Inspection Service (FSIS) maintains jurisdiction over meat and poultry products, whereas the FDA regulates other areas of food processing. Facilities incorporating robotic solutions often need to coordinate with both agencies when designing cleaning routines and validating process controls. For example, suppose a cobot is responsible for the automated cutting and portioning of raw poultry. In that case, the facility must comply with USDA-FSIS guidelines (FSIS) regarding microbial sampling and daily verification of sanitation processes.

Recent industry practice demonstrates that successful reconciliation of these requirements often leverages third-party certification schemes, such as those guided by the European Hygienic Engineering & Design Group (EHEDG), 3-A Sanitary Standards, or NSF International certifications, which create a framework for harmonizing worker safety (per OSHA) and hygienic design (per USDA-FSIS) requirements. For example, processing plants have implemented engineering controls and hygienic machine guarding systems that meet IP69K ingress protection and are explicitly designed for tool-less cleaning and inspection, thus satisfying both easy-clean mandates and the need for robust guarding against accidental mechanical contact. Adopting these certified housings and guards helps streamline dual-agency compliance and has been widely adopted as a practical solution in modern automated lines [38].

Additionally, model coordination frameworks have emerged in the form of multi-agency task forces (sometimes involving state-level OSHA consultation offices, FSIS liaisons, and outside experts) or ongoing “joint review” committees during new equipment procurement, where all regulatory stakeholders participate in pre-approval and post-installation validation audits. In the U.S., facilities often refer to checklists or integration protocols developed by the Alliance for Advanced Sanitation and the North American Meat Institute, and to guidance consolidated in regional emphasis programs (e.g., OSHA Region VI REP for poultry processing) to ensure alignment between federal safety and food-contact cleanliness standards.

## 5. Technological Solutions for Safety in Cobots

Modern cobots provide a sophisticated toolkit of hardware and software features designed to simultaneously address worker and food safety requirements mentioned in the above section. These solutions are derived from interdisciplinary research in robotics, machine vision, materials science, and ergonomics.

### 5.1. Worker-Oriented Safety Features

#### 5.1.1. Power and Force Limiting

Power and force limiting allow a cobot to detect collisions via integrated torque sensors [39]. For instance, Universal Robots’ e-Series arms monitor joint torques in real-time and immediately halt motion if preset thresholds are exceeded. This feature is crucial for tasks such as portioning poultry cuts, which involve sharp tools and high processing speeds. If a worker accidentally intrudes on the robot’s path while trimming meat, the cobot’s built-in force feedback triggers an emergency stop to prevent harm.

#### 5.1.2. Dynamic Safety Zones and Speed Separation Monitoring

Robots in collaborative environments can be equipped with dynamic safety zones to protect human workers. These safety zones are defined areas around the robot where human workers are prohibited from entering while the robot is in operation. These safety zones ensure minimal contact between the robot and the human worker, reducing the risk of accidents or collisions [40,41]. Many cobot systems (e.g., KUKA’s LBR iiwa) employ proximity sensors or 3D vision to establish dynamic safety zones. Also, the robot’s operational speed is controlled by the distance to a human worker, slowing down or stopping as a person approaches [42,43,44]. This speed and separation monitoring system ensures the robot remains at a safe distance from the human worker. So, when an operator approaches the dynamic safety zone, the robot’s speed and range of motion automatically scale down. Such adaptive speed and separation monitoring are particularly relevant in high-density poultry lines where multiple operators and robots share cramped workstations. If the sensor detects an unexpected movement near the blade or gripper, the robot transitions into a slower, “collaborative” mode, aligned with the thresholds set by ISO/TS 15066.

#### 5.1.3. Emergency Stop and Redundant Safety Layers

Cobots typically include both hardware E-stop buttons and software-based overrides. In high-risk environments such as poultry cutting stations, dual-channel redundancy ensures that the other channel remains active if a single-channel circuit fails [41]. This redundancy aligns with ISO 13849-1 requirements for safety-related parts of control systems (ISO). Training programs instruct staff on immediate E-stop procedures, underscoring OSHA’s emphasis on hazard recognition [45].

### 5.2. Sanitation-Oriented Safety Features

#### 5.2.1. Hygienic Design and Food-Grade Materials

Hygienic design principles mandate smooth surfaces, minimal crevices, and resistance to corrosive cleaning agents (OSHA). Cobots intended for direct or indirect contact with poultry may use stainless steel or food-grade polymer coatings, sealed gaskets, and shielded wiring. Such a design minimizes harborage points for bacteria, facilitating thorough washdowns. Some models feature quick-release end-effectors, allowing cutting blades or grippers to be removed and sanitized separately, thereby streamlining SSOP compliance.

#### 5.2.2. Automated Cleaning and Sensor Integration

Advanced cobot solutions integrate in-line cleaning. For example, nozzles built into the workstation can periodically spray disinfectants to ensure the end-effector and conveyor remain free from pathogens. Optical sensors or UV-based microbial detection systems can be mounted on or near the robot to identify real-time contamination hotspots [46]. This approach is especially valuable in preventing *Salmonella* cross-contamination during evisceration or deboning, as any detected increase in bacterial load can trigger automated alerts or halt production, thereby maintaining HACCP standards.

## 6. Psychological, Social, and Cultural Dimensions of Human–Robot Collaboration

While much research on human–robot collaboration (HRC) emphasizes mechanical precision, safety systems, and productivity, an expanding body of literature highlights the importance of psychological, social, and cultural factors in determining whether these technologies are successfully integrated into human work environments. Psychological constructs such as “trust”, “perceived safety”, and “mental workload” are critical determinants of operator acceptance and effective cooperation. Trust has emerged as a central mediator between system design and user behavior: operators who perceive a cobot as reliable and predictable are more likely to engage safely and confidently with it [47,48]. Likewise, excessive cognitive or emotional workload can reduce coordination fluency and increase error rates, underscoring the need to balance automation with human cognitive capacities [49].

Social and organizational dimensions further shape collaborative outcomes. Human–cobot “team fluency”, the perceived smoothness and synchrony of interaction, depends on clear role definition, mutual adaptation, and transparent communication cues [50]. Studies drawing on social psychology suggest that workers interpret robots through social identity frameworks, negotiating roles and boundaries much as they would with human teammates [51]. The introduction of cobots can therefore affect not only task performance but also group dynamics, job satisfaction, and workers’ sense of autonomy and competence [52].

Cultural perspectives add yet another layer of complexity. Cross-cultural research demonstrates that attitudes toward robots, preferred interaction styles, and tolerance for anthropomorphic features vary widely across societies [53,54]. As a result, uniform deployment strategies risk overlooking local values, communication norms, and conceptions of “helpful” behavior.

Collectively, this literature calls for an “integrated socio-technical approach” to HRC that measures both human and system variables. Incorporating validated instruments, such as trust and safety perception scales, the NASA Task Load Index (TLX) method assesses workload on five 7-point scales. Increments of high, medium, and low. NASA-TLX for workload, and team fluency questionnaires, alongside qualitative feedback, can help organizations design cobot systems that are not only efficient but also psychologically sustainable and culturally appropriate for their workforce.

## 7. Case Studies and Implementation

Several poultry plants have successfully deployed collaborative robots (cobots) for advanced processing tasks, achieving notable improvements in safety, yield, and efficiency

### 7.1. SINTEF & DENSO Gribbot (Norway)

At SINTEF, engineers developed the Gribbot, a cobot powered by the DENSO VS-087 robot, to automate the extraction of chicken fillets. The system leverages machine vision to locate gripping points, enabling precise scraping of fillets, a task historically performed by humans due to high variability and slippery surfaces. This deployment demonstrated that robots can achieve yields comparable to skilled human operators, opening new automation possibilities for food manufacturers [55].

### 7.2. Georgia Tech Robotic Deboning Line (USA)

Georgia Tech collaborated with poultry producers to create a robotized deboning line. These cobots use advanced cameras and AI to dynamically tailor knife paths for each chicken, maximizing meat yield and reducing waste. Unlike traditional fixed automation, these flexible systems can adapt to carcass variation, resulting in higher efficiency and less meat left on bones compared to older solutions [11].

### 7.3. Robotic Workbenches (USA)

A series of robotic workbenches are being utilized to handle tasks such as rehang, deboning, and cone loading. Each station is equipped with sensory tools and cobots to manage poultry pieces individually, a modular, scalable approach that further enhances yield and labor efficiency [56].

### 7.4. Human-Robot Collaboration Training (Arkansas)

Research at the University of Arkansas found that focused, hands-on training programs led workers to feel safer and more trusting of cobots in poultry processing environments, thereby increasing the adoption success rate of technology and the effectiveness of human-robot partnerships.

### 7.5. Sanitation

The Center for Scalable and Intelligent Automation in Poultry Processing aims to explore robotic and sensor-assisted sanitation, while also assessing the social impacts of such technology on the industry and its surrounding communities [8]. An automated vehicle equipped with hoses and scrubbers can be used to clean around a poultry plant. Another plan is to use a mobile robotic platform equipped with a biosensor to “biomap” the facility, highlighting areas with high concentrations of organic material or bacterial populations. When the robot finds a hot spot, it takes a swab sample for a traditional microbial analysis. The team is also developing a hyperspectral imaging system to detect foreign materials on the processing line, including plastic pieces, gloves, and other non-metallic items that X-rays cannot detect.

In summary, the integration of collaborative robots into poultry processing plants represents a paradigm shift within the industry, moving beyond basic automation toward intelligent, adaptive solutions that enhance technological efficiency and empower human labor. These case studies exemplify how strategic partnerships between engineers, researchers, and operators not only drive improved yields and operational flexibility but also foster safer, more collaborative workplaces where workers and cobots can thrive together. As adoption expands and systems become increasingly modular and customizable, the lessons learned from these pioneering implementations will continue to shape the future of food manufacturing, enabling greater productivity, sustainability, and workplace satisfaction.

## 8. Benefits of HRC Integration

### 8.1. Worker Safety

Human-robot collaboration (HRC) is increasingly recognized as a transformative approach to improving safety, efficiency, and overall working conditions in poultry processing plants. Integrating cobots with human workers offers several key benefits that directly address the safety challenges inherent in these demanding environments. Cobots can take over the repetitive, physically taxing tasks such as lifting, cutting, and handling birds, reducing musculoskeletal disorders and repetitive strain injuries for human workers. The cobot allows human workers to focus on more complex supervisory or decision-making tasks, reducing the risk of injuries and long-term health issues [21,57].

### 8.2. Food Safety and Quality

Human-robot collaboration leverages the strengths of both parties: robots provide precision, consistency, and data-driven insights, while humans oversee complex decision-making and adapt to nuanced situations. Automation minimizes direct human contact with poultry products, significantly lowering the risk of contamination from pathogens and foreign objects. This is particularly crucial in processing plants, where hygiene is paramount [58].

Advanced robotic systems equipped with biosensors and hyperspectral imaging can detect bacterial and viral pathogens in real time. These systems enable early intervention, such as isolating infected birds or removing contaminated products, thus preventing outbreaks and ensuring safer food [59]. Robots can autonomously collect environmental swabs, map sanitation efficiency, and monitor for contamination hotspots. This proactive approach rapidly responds to emerging food safety threats and helps optimize sanitization protocols [59]. Automated vision systems and sensors inspect poultry for defects, contaminants, and proper sizing, ensuring that only high-quality products reach consumers. These systems provide real-time detection and can be seamlessly integrated into existing production lines, reducing human error [60].

Several peer-reviewed and industry studies have quantified the reduction in repetitive strain injuries (RSIs) after the deployment of collaborative robots (cobots) in industrial and food sector environments:

• A 2025 industry case review reported by Farrelly Mitchell details a food manufacturer’s experience after introducing cobots for palletizing operations: injury-related downtime and workers’ compensation claims were reduced by 40% within the first year of cobot deployment, with these reductions explicitly attributed to the alleviation of repetitive, high-risk manual tasks [58].
• A 2023 systematic review by Lorenzini et al. [61] synthesizes multiple peer-reviewed sources, showing that collaborative robots, when incorporated into workstations, directly reduce biomechanical overload, physical strain, and cumulative trauma risks as measured by ergonomic assessment tools. This paper references specific interventions in which musculoskeletal disorder (MSD) and RSI incidence rates dropped significantly after cobot introduction.
• A 2025 article by Liu et al. [62] reports quantitative evaluations of ergonomic improvement and physical strain reduction from HRC systems, with documented decreases in work-related musculoskeletal injury risks after cobot adoption in manufacturing and assembly.
• A field-specific ergonomic intervention case compiled by Wired Workers and cited in food manufacturing settings demonstrates reduced rates of repetitive motion injuries, reduced absenteeism, and improved productivity after the integration of cobots for material handling and packaging [63].

While some of these studies aggregate industrial settings, not all are exclusive to poultry processing, and their findings are widely cited as direct analogues and as support for expected RSI-reduction outcomes following cobot deployment in meat and poultry processing tasks.

## 9. Challenges and Future Directions

Integrating worker safety and food safety in poultry processing through HRC faces several notable research gaps. These gaps span technical, human factors, regulatory, and adoption domains, reflecting the complexity of deploying collaborative robots in dynamic food environments while ensuring dual safety goals for both product and personnel [64].

### 9.1. Technical and Process Adaptation Gaps

Current automation systems struggle with the inherent product variability in poultry, making consistent, safe processing and contaminant control difficult [56]. Most available robotic solutions are highly specialized, inflexible, and costly, and lack multipurpose, adaptable platforms that could enhance both worker and food safety across tasks [65]. Sensing technologies capable of ensuring precise cuts and robust food safety interventions during variable product inflow are underdeveloped and have only recently seen integrated advancements, such as real-time collision detection and X-ray sensing [56].

### 9.2. Human Factors and Training Challenges

Familiarity, trust, and acceptance among workers are significant barriers, especially regarding concerns about physical safety and perceived job displacement [66]. Targeted, hands-on training can reduce safety concerns, but recruitment for such studies remains limited; generalizable, scalable workforce education strategies are lacking [64]. Effective frameworks for safety and transparency need worker-centric feedback to ensure that humans are appropriately engaged, aware of robot actions, and can intervene when necessary—there is limited empirical study on optimal feedback loops and shared control mechanisms [65].

### 9.3. Food Safety and Contamination Control

Although robots can reduce direct human contact (and thus cross-contamination risks), monitoring and validation methods to ensure food safety in mixed human-robot workflows are insufficiently developed and under-researched [65]. There are gaps in validating automated systems’ ability to maintain or exceed regulatory thresholds for hygiene and pathogen controls when deployed alongside humans [8].

### 9.4. Regulatory and Implementation Barriers

Regulatory standards governing collaborative robots in food settings are still evolving, leading to uncertainty and inconsistent practices regarding both worker and food safety [64]. Industry expert surveys reveal that while engineering feasibility is progressing, real-world adoption lags due to unclear guidelines, liability concerns, and the cost of upgrading infrastructure for collaborative automation [66].

Retrofitting existing facilities originally designed for manual labor is complex and costly. Infrastructural changes may be needed to accommodate mobile robots, collaborative work cells, or vision-guided automation.

### 9.5. End Effectors

Suction cups are low-cost and widely used, but they lose seal reliability on wet, slimy, porous, or irregular poultry surfaces, which is why many tasks still depend on humans in plants. Moisture, surface curvature near joints, feather follicles/pores, and variable skin compliance make vacuum maintenance difficult, leading to drops or product damage and restricting automation at the key cut/transfer step.

#### 9.5.1. Food-Grade Soft/Elastomer Grippers

Compliant finger grippers using FDA/EU food-contact elastomers (e.g., silicone or TPU) distribute contact pressure to avoid bruising skin and tearing fascia, while conforming to variable contours at thighs, wings, and keel areas [67]. Design priorities include minimal crevices, quick-release tooling, chemical resistance to peracetic acid/quat foams, and high-pressure washdown compatibility, in line with EHEDG hygienic design principles to reduce bacterial harborage [68]. Reviews of food end-effector gaps underscore the need for a few mechanical parts to avoid component loss into the product, achieve low cost, and ensure compliance with washdown, which current soft-gripper programs explicitly target in poultry prototypes.

#### 9.5.2. Adaptive Suction and Vacuum Innovations

Adaptive suction systems pair multi-bellows food-grade cups with deformable lips and local compliance frames to maintain seals across curved, slippery poultry surfaces, improving pick stability compared to rigid cups in wet conditions [25]. Multichannel vacuum with flow-sensing can detect partial seals and trigger regrasp or backup grasp points; this integrates with HACCP/SSOP-informed cleaning, including inline nozzle rinses of cup lips between picks to prevent biofilm buildup on contact edges [25]. Hybrid finger-plus-suction designs use light suction for stabilization, with compliant pads providing the primary load path, limiting peak skin shear while reducing drop rates during evisceration transfer and post-chill singulation.

#### 9.5.3. Bio-Inspired and Fabric/Pneumatic Approaches

Underactuated, tendon-driven fingers and origami/pneumatic networks provide shape adaptation with few moving parts, addressing the “soft, uneven, non-uniform” constraint highlighted for poultry and reducing the risk of part detachment into food streams [69]. Fabric-reinforced pneumatic actuators and granular jamming pouches can envelop irregular cuts without concentrating pressure points, useful for deboned portions where suction fails; materials are being selected/treated for food contact and washdown durability per EHEDG and SSOP needs [25]. Grasp planners trained on variable carcass geometry integrated vision and force cues to select contact regions with lower contamination risk and better sealability, aligning with the review’s call for AI that handles irregular shapes under uncontrolled plant lighting and moisture [70].

#### 9.5.4. Sanitation, Materials, and Washdown

End-effectors increasingly use stainless or coated housings, sealed gaskets, and smooth surfaces; soft elements are modular for rapid tool-less removal and for COP/CIP, matching SSOPs that specify hot-water rinses, alkaline foams, and disinfectants common in poultry lines [25]. Designs target IP69K-style ingress protection at the tool body and compatible chemistry at the contact interface to withstand repeated high-pressure, high-temperature cleaning cycles without swelling, cracking, or loss of frictional properties.

#### 9.5.5. Control and Sensing for Gentle Handling

Closed-loop force/torque limits from cobots are paired with fingertip pressure sensing and vision to prevent bruising and skin tearing; this marries ISO/TS 15066 force ceilings with poultry-specific quality protection during cuts and transfers. Dynamic speed/separation monitoring and emergency-stop redundancy are maintained even with soft tooling, ensuring worker safety when operators co-manipulate carcasses during semi-automated trimming or regrasp events.

Research in biomedical engineering and food automation has explored integrating flexible force sensors (FSRs) and variable-angle mechanisms into forceps for grasping and manipulating various tissue samples, including chicken meat. These adaptable tools can accommodate different shapes and textures, such as cooked versus raw meat, by adjusting grip force and jaw alignment. This approach offers improved handling precision and reduced tissue damage, which is critical for poultry lines where anatomical variation is common [25].

### 9.6. Computer Vision for Disease Detection

Recent advances in machine learning and deep learning have enabled the deployment of computer vision systems for disease detection in production-line environments. These systems can analyze fecal images, bird postures, and behavioral patterns in real time to identify diseases such as coccidiosis and *Salmonella*. For example, new YOLO (You Only Look Once)-based detectors and EfficientNet classifiers have demonstrated fast, accurate identification of disease states in poultry using image data collected from commercial farms. Such systems accommodate variable lighting, occlusions, and the dynamic movement of birds in high-throughput settings, directly addressing the challenges of real-time production environments [71].

### 9.7. Cost and ROI

High initial costs for robotic solutions, especially for small and mid-sized companies, can deter investment. Uncertain payback periods and the need for regular updates and maintenance also challenge the economic case for adoption. The cost of a collaborative robot in poultry processing typically ranges from $25,000 to $75,000, with a rapid return on investment (ROI) often achieved within 1 to 2 years. ROI is driven by increased productivity, reduced labor costs (potentially by 25–40%), fewer errors, improved product quality, and enhanced worker safety, leading to faster cycle times and 24/7 operations [72].

### 9.8. Future Research Directions

Multidisciplinary work linking robotics, occupational health, and food safety science is needed to create holistic, evidence-based intervention models. Studies on scalable training, feedback integration, and human-robot workflow optimization will be crucial for safe, efficient adoption. Validation studies benchmarking microbial control, worker injury risks, and overall risk mitigation in hybrid environments are especially limited and warrant systematic investigation to establish best practices.

The gap between promising lab results and practical, scalable industrial solutions remains wide. Collaboration between academic researchers, technology developers, and poultry processors is crucial to ensure real-world applicability [73].

In summary, bridging these gaps will require advancing flexible automation, rigorous human factors research, improved sensing and safety feedback systems, and the development of adaptive regulatory frameworks to enable the safe, productive, and hygienic integration of collaborative robots within poultry processing.

## 10. Multidisciplinary Roadmap for Integrating Worker and Food Safety in Poultry Processing

To address the persistent gaps in collaborative robotics integration, this roadmap leverages interdisciplinary research and field-tested solutions in robotics, sanitary engineering, human factors, and regulatory affairs. The following actions are designed to foster safe, resilient, and ethically sound poultry processing operations.

### 10.1. Harmonize Safety and Hygiene Standards

• Launch joint working groups of robotics engineers, food technologists, and regulatory experts to create unified compliance protocols for cobot deployment (e.g., EHEDG and ISOTS 15066).
• Pilot cross-standard checklists in specific plants, merging force-limiting, microbial control, and ergonomic requirements into single audit frameworks.
• Schedule regular audits led by interdisciplinary teams to ensure both mechanical and microbial safety are verified with robust data collection and traceability.

### 10.2. Advanced Ergonomic Design and Human Factors

• Integrate digital simulations and VR-based pre-validation to optimize workspace layouts, minimizing repetitive motion injuries and enhancing interface usability.
• Specify the installation of height-adjustable workstations, anti-fatigue surfaces, and real-time ergonomic monitoring devices.
• Co-design cobot workflows with human factors specialists and frontline workers to ensure both safety and acceptance, adjusting systems based on measured well-being indices.

### 10.3. Workforce Training and Change Management

• Develop training curricula that blend technical (robot operation, safety protocols) and sanitation (SSOP, HACCP) content, tailored for multilingual and multilevel workforce profiles.
• Implement hands-on and VR-based demonstration sessions, using feedback loops (surveys, committees) to adapt programs in response to user barriers and acceptance trends.
• Establish mentorship and champion programs to support rapid staff upskilling and foster a positive culture around technology adoption.

### 10.4. Data-Driven Monitoring and Predictive Analytics

• Integrate the Industrial Internet of Things (IIoT), thermal cameras, and microbial biosensors for real-time tracking of robot performance, workplace hygiene, and contamination risks.
• Use predictive analytics to schedule preventive maintenance for robotic joints/end-effectors and trigger automated alerts when hygiene thresholds are breached.
• Set up continuous process monitoring dashboards, enabling instant intervention and documenting each improvement or incident.

### 10.5. Research and Innovation Targets

• Prioritize explainable AI systems for robotic food handling, enabling transparent and actionable insights for front-line users.
• Develop and publish standardized datasets for poultry processing that account for regional product variability, with explicit labelling for process audit and regulatory harmonization.
• Support cross-sector academic-industry partnerships to accelerate prototype development and validate new ergonomic, sensor, and sanitation solutions.

### 10.6. Implementation of Benchmarks and Feedback

• Define measurable KPIs (worker injuries, contamination events, process throughput, technology acceptance rates) to gauge roadmap effect and guide iterative improvement.
• Establish a collaborative stakeholder forum, collecting regular feedback and sharing best practices, case studies, and outcomes.

## 11. Conclusions

This comprehensive review demonstrates that the successful integration of collaborative robots in poultry processing hinges on aligning worker safety and food safety, leveraging advances in robotics, hygienic design, and human factors research. Human-robot collaboration (HRC) offers measurable improvements in reducing occupational injury rates and limiting contamination, especially when adaptive sensing, soft grippers, and vision-based quality control are employed for handling non-uniform poultry products. Addressing social and psychological dimensions, including trust, training, and cross-cultural perspectives, is critical to worker acceptance and long-term technology adoption in real-world food environments.

Despite this technology’s potential, persistent challenges remain. Current limitations include the adaptability of grippers and sensors for wet, irregular items; incomplete regulatory frameworks and inconsistent best practices for cobot deployment; barriers to workforce retraining; and gaps in microbiological validation and feedback monitoring in mixed robot-human workflows. Economic feasibility also poses hurdles for small and mid-sized facilities, though evidence suggests that rapid return on investment can be achieved with proper implementation and training, alongside reduced compensation claims due to decreased repetitive injuries.

A multidisciplinary roadmap is needed to bridge these gaps, calling for collaborative action between industry, researchers, and regulatory agencies to harmonize ergonomic, safety, and hygiene standards. Key priorities include the development of explainable AI, adaptive, food-grade end-effectors, standardized datasets for training and monitoring, and holistic education programs tailored for diverse workforces. Ongoing evaluation through measurable key performance indicators (KPIs) and feedback loops will help ensure both safety and productivity, driving sustainable food operations and consumer confidence.

In summary, the path toward resilient, efficient, and hygienic poultry processing lies in integrating advanced robotics with robust human factors design and proactive regulatory coordination. These efforts will empower the food industry to deliver safe products at scale, while safeguarding both the workforce and public health, heralding a new era for human-robot collaboration in food production.

## Figures and Tables

**Table 1:** Comparison of international standards on robotics.

	ISO 10218 [30]	ISO/TS 15066 [32]	ANSI/RIA R15.06 [33]
Scope	Industrial robots and robot systems	Collaborative robot	The U.S. national adoption of the ISO 10218 standard.
Key aspects	Covers robot design, risk assessment, functional safety, safeguarding, and information for use.	Human-robot interaction/collaboration (safety-rated monitored stop, hand-guiding, speed and separation monitoring, and power and force limiting)	Safe manufacture, installation, integration, and safeguarding of industrial robots and robot systems.
Exclusions	Non-industrial robots and service robots	Not standalone, supports ISO 10218	Non-industrial robots: U.S. focused only

## Data Availability

No new data were created or analyzed in this study. Data sharing is not applicable.
